# Graphene Based Poly(Vinyl Alcohol) Nanocomposites Prepared by In Situ Green Reduction of Graphene Oxide by Ascorbic Acid: Influence of Graphene Content and Glycerol Plasticizer on Properties

**DOI:** 10.3390/nano8121013

**Published:** 2018-12-06

**Authors:** Mónica Cobos, M. Jesús Fernández, M. Dolores Fernández

**Affiliations:** Department of Polymer Science and Technology. Faculty of Chemistry, University of the Basque Country UPV/EHU, Paseo Manuel Lardizábal 3, 20018 San Sebastián, Spain; monica.cobos@ehu.es (M.C.); mariadolores.fernandez@ehu.es (M.D.F.)

**Keywords:** graphene, poly(vinyl alcohol), glycerol, nanocomposites, graphene oxide green reduction

## Abstract

The enhanced properties of polymer nanocomposites as compared with pure polymers are only achieved in the presence of well-dispersed nanofillers and strong interfacial adhesion. In this study, we report the preparation of nanocomposite films based on poly(vinyl alcohol) (PVA) filled with well dispersed graphene sheets (GS) by in situ reduction of graphene oxide (GO) dispersed in PVA solution using ascorbic acid (L-AA) as environmentally friendly reductant. The combined effect of GS content and glycerol as plasticizer on the structure, thermal, mechanical, water absorption, and water barrier properties of PVA/GS nanocomposite films is studied for the first time. Higher glass transition temperature, lower crystallinity, melting, and crystallization temperature, higher mechanical properties, and remarkable improvement in the thermal stability compared to neat PVA are obtained as a result of strong interfacial interactions between GS and PVA by hydrogen bonding. PVA/GS composite film prepared by ex situ process is more brittle than its in situ prepared counterpart. The presence of GS improves the water barrier and water resistance properties of nanocomposite films by decreasing water vapor permeability and water absorption of PVA. This work demonstrates that the tailoring of PVA/GS nanocomposite properties is enabled by controlling GS and glycerol content. The new developed materials, particularly those containing plasticizer, could be potential carriers for transdermal drug delivery.

## 1. Introduction

Poly(vinyl alcohol) (PVA) is a water-soluble semi-crystalline synthetic polymer obtained from the precursor polymer poly(vinyl acetate). Based on its excellent chemical, physical, mechanical, and thermal properties, outstanding film-forming ability, biodegradability, biocompatibility and non-toxicity [1,2,3], PVA has many uses such as adhesives, coatings, films, membranes, drug delivery systems and fuel cells, that have been applied in the industrial, commercial, medical, and food fields [4,5,6,7]. Owing to the strong inter and intramolecular hydrogen bonds between hydroxyl groups, PVA has a high melting point that is close to its decomposition temperature which makes its melt processing very difficult and, hence, that PVA has been processed mainly from aqueous solutions. Moreover, PVA films are too brittle because of high crystallinity. To overcome these drawbacks plasticizers have been incorporated into PVA [1,8,9,10,11]. Although water is the most effective plasticizer for PVA, glycerol has been widely used due to its low toxicity, environmental friendliness, and its low vapor pressure compared to water or other polyols such as ethylene glycol. Plasticizers form strong hydrogen bonding with the hydroxyl groups of PVA and reduce the inter and intra-molecular hydrogen bonding between PVA chains, which leads to an increase of free volume and chain movements, reducing the melting point, improving flexibility, and handling of films and preventing cracks in the polymeric matrix.

The dispersion of nanoscale fillers (ranging from 0.1 nm to 100 nm) is the characteristic feature of polymer nanocomposites. These new composite materials have attracted great interest in the last decade since a synergy between fillers and polymer chains can be created and, thus, materials with enhanced properties can be obtained [12,13]. These improved properties can only be achieved if the nanofiller is well dispersed in the polymer matrix. Among the nanofillers graphene is being extensively used to prepared polymer nanocomposites due to its unique properties (high mechanical strength, high aspect ratio, high electrical and thermal conductivity, high gas impermeability, and low density), and the natural abundance and inexpensive cost of its graphite precursor [14,15,16,17,18]. Graphene, the two-dimensional allotrope of carbon, is a one-atom-thick planar sheet of sp^2^ bonded carbon atoms densely packaged in a honeycomb crystal. The most popular way to produce graphene sheets on a large area is from the graphite oxide precursor, followed by its exfoliation and reduction [19,20]. The exfoliation of graphite oxide, usually achieved via ultrasonic treatment of graphite oxide dispersions, results in the separation of graphene layers and the formation of graphene oxide (GO) sheets [21,22]. GO can be converted to graphene by thermal, electrochemical and chemical reduction methods [23,24,25]. The removal of the oxygen functionalities (hydroxyl, carbonyl, and alkoxy groups) on graphene oxide has been achieved by using various toxic, corrosive, and hazardous chemical reagents (hydrazine and its derivatives, sodium borohydride, hydroiodic acid). Among the developed alternative green reducing agents for GO, the ascorbic acid is considered an ideal substitute for the most efficient reductant (hydrazine) [26,27]. Another effective method for obtaining graphene is the non-chemical reduction of GO hydrothermally and solvothermally [28,29,30,31,32].

The properties of polymer nanocomposites are highly related to the microstructures, and uniform dispersion of nanofiller and absence of agglomerates in the polymer matrix is required to achieve an improvement in the properties. During the reduction process GO sheets become more hydrophobic and tends to agglomerate rapidly into flakes of monolayered sheets, to restack to form graphite and precipitate. The in situ reduction of GO dispersed in a polymer matrix is one of the most effective strategies employed to accomplish stable dispersions of graphene [33]. The main strategies for preparation of polymer/graphene nanocomposites are in situ intercalative polymerization, solution, and melt intercalation. The solution casting technique is the most straightforward method, and compared with the melt mixing is more effective to achieve good dispersity and distribution of the graphene in the polymer matrix [34].

The incorporation of graphene and its derivatives in PVA could bring remarkable enhancements in its properties and broaden its applications. Various graphene derivatives have been used in the preparation of PVA nanocomposites, and enhancement in the bulk physical properties has been reported. There are some studies reported in the literature on PVA/graphene nanocomposites prepared by reduction of GO in presence of PVA [35,36,37,38,39,40,41,42,43,44,45,46,47].

To the best of our knowledge, up to now neither the preparation of PVA/graphene nanocomposites by in situ reduction of GO with ascorbic acid nor the effect of incorporation of plasticizer on the properties of the composites has been addressed in the literature. In this study we show a simple, efficient and environment-friendly method to prepare PVA/graphene nanocomposites by in situ reduction of GO dispersed in PVA water solution, using ascorbic acid as the reducing reagent. In addition, the effect of addition of glycerol as plasticizer and the graphene content on the thermal, mechanical, water absorption, and water barrier properties of the nanocomposites is investigated followed by the structural and morphological characterization of the nanocomposites by different techniques. Additionally, a comparative study of an analogous PVA/graphene nanocomposite film prepared by the ex situ method is also reported. This green method has several advantages: its environmental friendliness, the most abundant and smallest graphitic domains produced by ascorbic acid as compared to other environment-friendly reductants [48,49], the suitability of the ascorbic acid reduced GO for biomedical applications, since both the L-AA and the resulting oxidized products are eco-friendly, and the prevention of graphene agglomeration in the PVA matrix. In this study, we have demonstrated that, by controlling the nanofiller and glycerol plasticizer content, the properties of PVA/GS nanocomposites may be tailored.

## 2. Experimental Section

### 2.1. Materials 

Graphite flakes were purchased from Alfa Aesar (Karlsruhe, Germany) (99.8%, 325 mesh), poly(vinyl alcohol) (PVA) (Mw= 61,000 Da; degree of hydrolysis 98.0–98.8 mol %), glycerol and l-ascorbic acid were supplied by Sigma-Aldrich (Munich, Germany), sodium nitrate (NaNO_3_) was obtained from Merck (Darmstadt, Germany), while sulphuric acid (H_2_SO_4_, 98%), potassium permanganate (KMnO_4_), hydrogen peroxide (H_2_O_2_, 30 wt% aq.), and hydrochloric acid (HCl, 37% aq.) were acquired from Panreac (Barcelona, Spain). All chemicals were used as received without further purification.

### 2.2. Preparation of Graphite Oxide (GO) and Chemically Reduced Graphene Sheets (GS)

GO was synthesized from natural graphite powder by the modified Hummers method [50]. Details of the synthesis were described in our previous work [51]. To obtain GS, exfoliated graphene oxide sheets by ultrasonication were reduced by ascorbic acid maintaining the weight relationship between the L-AA and the GO fixed at 3.5, as described elsewhere [52].

### 2.3. Synthesis of PVA/Graphene Sheets Nanocomposite Films (PVA/GS) by the in Situ Method

Graphene oxide sheets were achieved by treatment of 30 mL of GO aqueous suspensions with a tip sonicator for 15 min. Separately, a 5 wt% aqueous solution of PVA was prepared at 100 °C upon stirring for 1 h, and subsequently cooled to room temperature. The suspension of graphene oxide sheets was then added to PVA solution while stirring. After being stirred overnight, the mixture was heated at 60 °C using an oil bath, then the desired amount of a water solution of L-AA was added (ratio of L-AA to GO was 3.5) under vigorous stirring. To reduce the graphene oxide sheets into graphene sheets (GS), the mixture was heated for 6 h with constant stirring and in the absence of light. To obtain free-standing PVA/GS films, the mixture was cast onto a plastic Petri dish and left to dry at room temperature. Prior to characterization, the films were dried under vacuum at 60 °C for three days and kept in desiccators. The same procedure was followed for the preparation of films containing plasticizer, by adding 20% weight glycerol to the PVA solution. The nanocomposites were denoted as PVA/GSx, where x indicates the weight percentage of GS.

### 2.4. Preparation of PVA/Graphene Sheets Nanocomposite Film by the ex Situ Method

PVA/graphene sheets nanocomposite film was prepared by blending PVA with chemically reduced GO. The desired amount of chemically reduced graphene oxide powder by L-AA was dispersed in 30 mL of deionized water by ultrasonication for 1 h, to get exfoliated graphene sheets. An aqueous solution of PVA (5 wt%) was prepared at 100 °C upon stirring for 1 h, and subsequently cooled to room temperature. The chemically reduced GO dispersion was then mixed with PVA solution and ultrasonicated for an additional 1 h. The film was prepared with 1 wt% of GS loading.

### 2.5. Characterization Techniques

X-ray photoelectron spectroscopy (XPS) was employed for the analysis of the surface chemistry of GO and GS, using a SPECS system equipped with a Phoibos 150 1D-DLD analyser (Berlin, Germany) and monochromatic Al Kα X-ray source (1486.6 eV). The XPS survey-scan spectra were recorded with pass energy of 80 eV, step energy 1 eV, and dwell time 0.1 s, whereas the individual high-resolution spectra were collected with pass energy of 30 eV, step energy 0.1 eV, and dwell time 0.1 s, at an electron take-off angle of 90°. A Renishaw Invia microscope (Gloucestershire, UK) with laser frequency of 514 nm was used to obtain the Raman spectra of the graphenic materials from 500 to 3500 cm^−1^. The information about the methods for the structural, morphological, microstructural, and thermal characterization of GO and GS is displayed in the Supplementary Material.

The XRD patterns of graphenic materials, PVA and its nanocomposites were performed on a Malvern Panalytical (Almelo, Netherlands) X’PERT PRO automatic diffractometer operating at 40 kV and 40 mA, in theta-theta configuration, secondary monochromator with Cu-Kα radiation (λ = 0.154 nm) and a PIXcel solid state detector (active length in 2*θ* 3.347°). Data were collected in the range of 2*θ* = 1–50° (step size of 0.026° and time per step of 80 s, total time 20 min) at room temperature. A variable divergence slit giving a constant 5 mm area of sample illumination was used. The Bragg equation (λ = 2*d* sin*θ*) was used to determine the interlayer distance in the graphenic materials.

A Hitachi S-4800 scanning electron microscope (Tokyo, Japan) operating at an accelerating voltage of 15 kV was used to obtain SEM images of the neat PVA and PVA/GS nanocomposite films, after being freeze fractured by liquid nitrogen and sputtered with gold. TEM micrographs of nanocomposites were obtained with a Philips Tecnai G2 20 TWIN TEM (Eindhoven, Netherlands) at 200 kV accelerated voltage after cutting the PVA/GS films into thin sections with a Leica EM UC6 ultramicrotome apparatus, at room temperature, and placing the sliced specimens in copper grids.

Differential scanning calorimetry analyses were performed by a Mettler Toledo DSC 3+ unit (Greifensee, Switzerland). The samples were heated from −30 °C to 250 °C at a heating rate of 10 °C/min under a nitrogen gas flow of 20 mL/min. Values were obtained from the first cooling and second heating scans.

Thermogravimetric analysis was performed on a TA instruments TG-Q-500 (New Castle, DE, USA) at a heating rate of 10 °C/min from 40 °C to 800 °C in nitrogen or air-flow.

An electromechanical testing machine (Instron 5967, Norwood, MA, USA) operating at room temperature with a load cell of 500 N, a gauge length of 10 mm, and a cross head speed of 5 mm/min was used to performed tensile tests. Films were were cut into a dog-bone shape before testing and kept at a relative humidity of 58% at room temperature for more than one week to ensure equilibration of the moisture uptake in the films. Testing was carried out on at least ten identical composite films of each composition and the average values were reported.

To measure the water absorption of unplasticized and plasticized PVA/GS nanocomposites, the samples were dried at 60°C for 24 h to a constant weight, cooled in a desiccator, and weighted (*W*_0_). The dried samples were immersed into distilled water maintained at 25 °C. They were removed after 24 h. The excess water on the swollen films surface was blotted with a filter paper, and the films were weighted (*W*_w_). Since PVA is soluble in hot water (95 °C) and slightly dissolved in cold water (room temperature), the films after immersion were dried again under vacuum for two days at 60 °C, and the weight was measured (*W*_d_). The total water absorbed (*W*_t_) by the sample was calculated by the following Equation (1):

(1) $$W_{t}\left(\%\right)=\frac{W_{w}-W_{d}}{W_{0}}\times \;100$$

Water vapor permeability (WVP) of unplasticized and plasticized nanocomposite films was determined according to the ASTM E96 standard, using the upright cup method. All dried film samples were conditioned for 1 week at 58% RH (relative humidity) and 25 °C before being analyzed. The films were sealed on cups containing deionized water and then the test cups were placed in an environmental chamber at 25 °C and 58% RH. Each cup was weighed to the nearest 0.00001 g on an electronic scale. The weight of each cup and the time were recorded. The weight of the cups was measured every 12 h up to 168 h after placement in the environmental chamber. Each sample was tested in triplicate. The water vapor transmission rate (WVTR) was calculated from the slope of the curve weight change as a function of time using linear regression analysis, according to Equation (2):

(2) $$\mathrm{WVTR}\;=\frac{slope\;\cdot \;\;d}{A\;\left(R_{1}-R_{2}\right)}$$

where *d* is the average thickness of the film, *A* the test area, *R*_1_ the relative vapor pressure in the permeation cell, and *R*_2_ the relative vapor pressure in the environmental chamber. The water vapor permeability (WVP) was calculated as:

(3) $$\mathrm{WVP}=\frac{\mathrm{WVTR}}{S}$$

where *S* is the saturation vapor pressure (Pa) of water at test temperature.

## 3. Results and Discussion

### 3.1. Characterization of GO and GS

**Figure 1:**
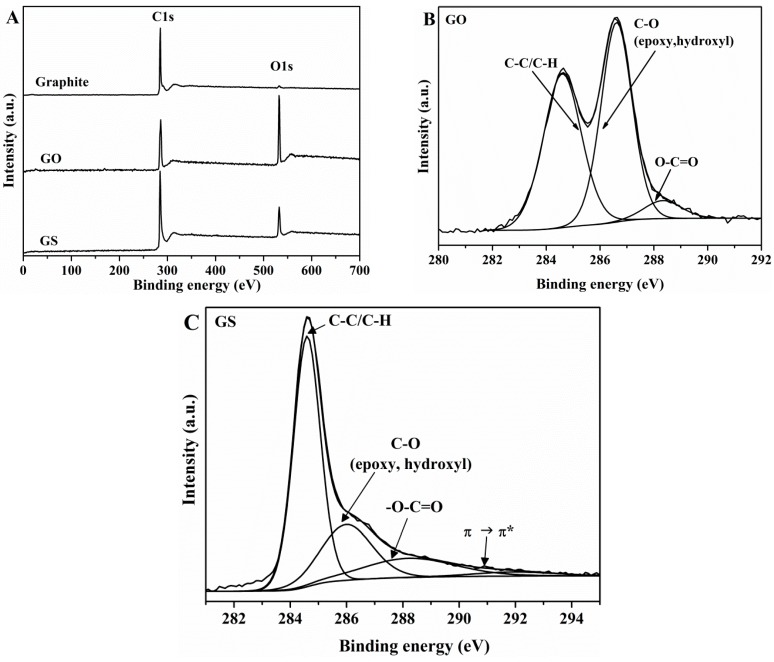
XPS survey (**A**,**B**), and (**C**) high resolution XPS spectra of natural graphite, GO, and GS.

In the XPS survey scan spectrum of GO (Figure 1A), it can be seen two intense peaks with binding energy of 284.6 and 532.9 eV, corresponding to C–C stretching and to O1s, respectively.

The high-resolution C1s XPS spectrum of GO (Figure 1B) exhibits the superposition of two strong peaks, the first at 284.6 eV assigned to C–C/C–H and the second one at 286.6 eV assigned to C–O (including epoxy and hydroxyl groups) bonds, and a third peak at 288.3 eV assigned to –O–C=O functional groups. This result suggests that highly-oxidized GO has been obtained, which agrees with previous reports [53]. The atomic percentage (at. %) for different carbon functional groups was calculated with respect to the total area of the C1s peak. The C/O atomic ratio in GO was 2.21. A narrower graphitic C–C signal (284.6 eV) is observed in the C1s spectrum of GS (Figure 1C) as compared with that of GO (FWHM value of 1.117 eV versus 1.665 eV), suggesting the development of a more homogeneous chemical environment and/or ordered graphitic structure. The oxygen functionalities assigned for GO can also be observed in the XPS spectrum of GS, while it can be seen a dramatic decrease in the intensity of the C–O peak (epoxy and alkoxy) which reveals that after reduction most oxygen functional groups have been removed. In addition, a new peak appears at 291.1 eV, due to π→π* transition of aromatic C=C bonds. The C/O atomic ratio increases from 2.21 for GO to 5.61 for GS. XPS results indicate that most of oxygen functional groups were removed.

**Figure 2:**
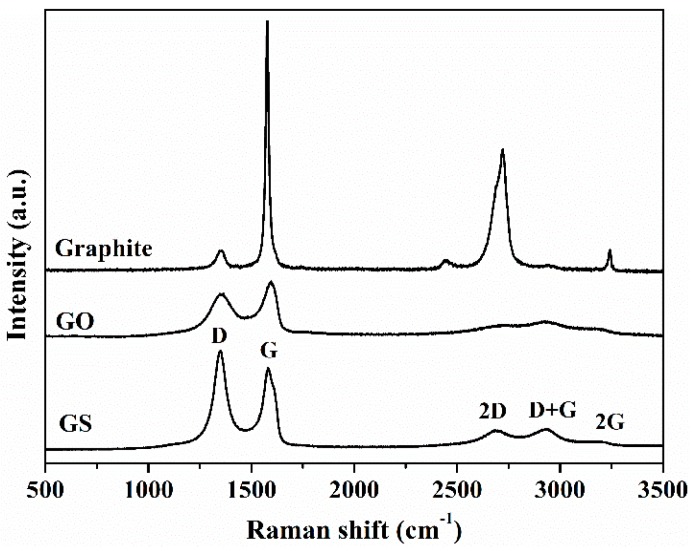
Raman spectra of natural graphite, GO, and GS.

Raman analysis reveals significant structural changes in graphene sheets during the oxidation of natural graphite and during the chemical reduction of GO. A noticeable change in the shape and intensity of the bands of GO is observed as compared to natural graphite (Figure 2). The G band, originated from the first order scattering of the E_2g_ phonon of sp^2^ C atoms [54,55], in the spectrum of GO is broadened and shifted towards a higher wavenumber, 1597 cm^−1^, due to the high oxidation level. The displacement of G band is associated with the presence of isolated double bonds [54,55]. The D band (1354 cm^−1^) due to the introduction of oxygen groups and other structural defects becomes broader and prominent. After oxidation a significant reduction in sp^2^ domains results in the broadening and the reduced intensity of the 2D band (2710 cm^−1^) [56,57]. In the spectrum of GO two new overtone bands appear at 2937 cm^−1^ and 3124 cm^−1^ which are denoted as D+G and 2G band, respectively, which relative intensity ratio (*I*_D_/*I*_G_) is a measure of disorder degree and is inversely proportional to the average size of the sp^2^ clusters [58]. The *I*_D_/*I*_G_ intensity ratios have been found to be 0.03 ± 0.02 and 0.78 ± 0.01 for natural graphite and GO, respectively. When comparing the Raman spectra of GS and GO, a shift to a lower wavenumber of the G band of GS is observed, indicating the recovery of the hexagonal network of carbon atoms with defects. In addition, a narrowed and more intense D band appears in the GS spectrum, indicating that structural defects are introduced. The *I*_D_/*I*_G_ intensity ratio for GS (1.23 ± 0.03) has been found to be larger than that for GO (0.78 ± 0.01), suggesting the formation of more sp^2^ domains. After reduction, the 2D band becomes more intense and defined. Several reports in the literature have shown that *I*_2D_/*I*_D+G_ is an indicator for the aromatic C-structural order of the graphitic materials, since the 2D mode is sensitive to the electronic structure in the graphene, whereas the D+G combination mode is induced by disorder [57]. The higher *I*_2D_/*I*_D+G_ ratio in GS (1.2 times higher than GO) indicates the restoration of graphitic electronic conjugation by L-AA reduction [54,59,60].

Results of structural, morphological, microstructural and thermal characterization of GO and GS by FTIR (Figure S1), XRD (Figure S2), SEM (Figure S3a), TEM (Figure S3b), and TGA (Figure S4), and its discussion are displayed in the Supplementary Material.

### 3.2. Characterization of PVA/GS Nanocomposites

Results of structural characterization of unplasticized and plasticized PVA/GS nanocomposites by FTIR (Figure S5), and its discussion are displayed in the Supplementary Material.

#### 3.2.1. X-ray Diffraction

**Figure 3:**
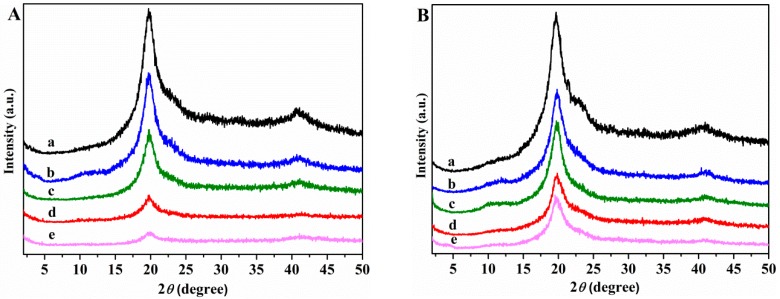
XRD patterns (**A**) (a) PVA; (b) PVA/GS0.5; (c) PVA/GS1; (d) PVA/GS1.5; (e) PVA/GS2; (**B**) (a) PVA/GLY; (b) PVA/GS0.5/GLY; (c) PVA/GS1/GLY; (d) PVA/GS1.5/GLY; and (e) PVA/GS2/GLY.

Figure 3A,B shows the X-ray diffraction spectra of neat unplasticized and plasticized PVA, and of nanocomposites in 2*θ* range 5°–50°.

The XRD patterns of neat unplasticized and glycerol plasticized PVA films show a main diffraction peak at 2*θ* = 19.9°, and another one at 2*θ* = 40.8° which correspond to (101) and (111) planes, respectively [61]. Compared with the unplasticized and glycerol plasticized PVA the nanocomposite samples show only the peaks at 2*θ* = 19.6° and 40.8°, while the broad peak of GS at 2*θ* = 24.3° disappears, indicating a homogeneous dispersion of GS sheets in the polymer matrix and not aggregated and restacked together. The intensity of the diffraction peak at 2*θ* = 19.9° decreases as the GS content increases, indicating a decrease of crystallinity of PVA. The reduced crystallinity of PVA after incorporation of GS within the unplasticized and glycerol plasticized PVA matrix can be due to the reduction in polymer chain mobility, that can be explained by the formation of a constrained polymer region [62]. This region located around the filler surface adjacent to the interface region exhibits different characteristics than those of neat polymer due to the interfacial interactions between the polymer matrix and the surface of the filler. The mobility of the polymer chains within this region is greatly hampered, and the degree of restriction of the mobility of the polymer chains is affected by the filler content [63]. On this basis, it may be inferred that the interactions through hydrogen bonding between residual oxygen functionalities of GS and the –OH groups of PVA restrict the polymer motion resulting in a dramatic decrease in the crystallinity of PVA in the presence of GS.

#### 3.2.2. Nanostructure and Morphology

**Figure 4:**
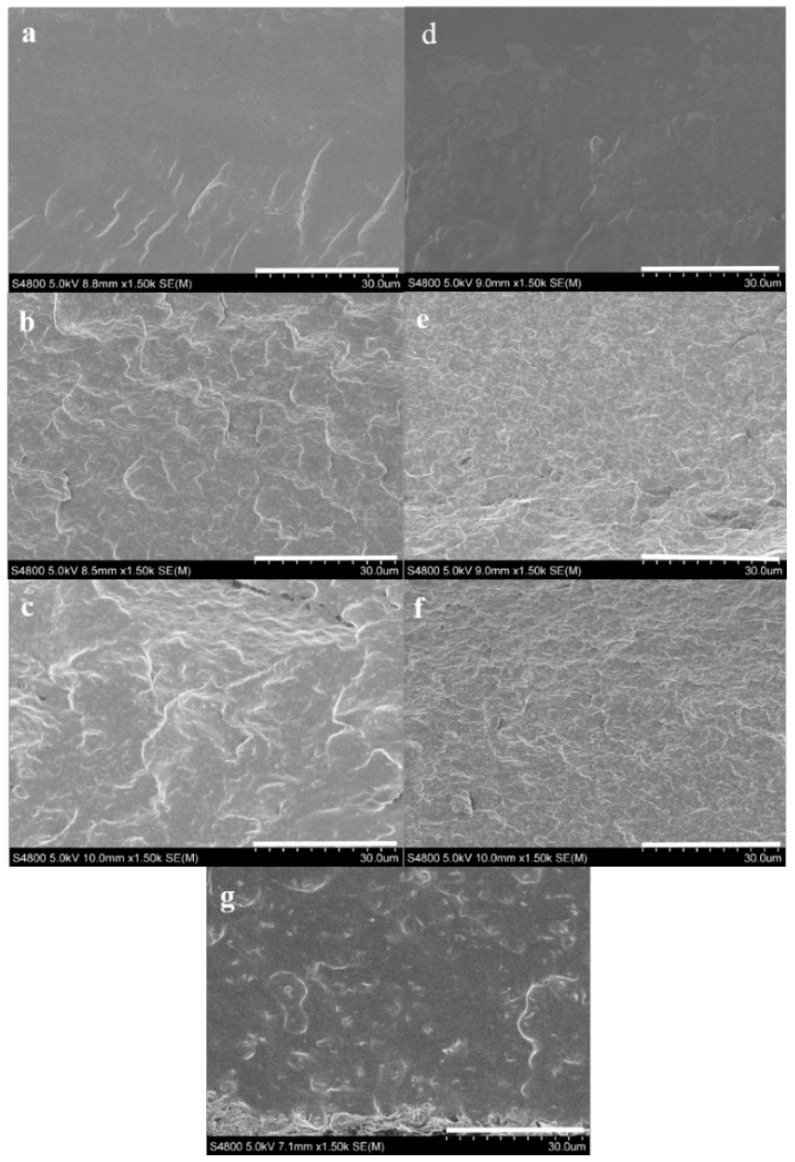
SEM images of the fractured surfaces of: (**a**) PVA; (**b**) PVA/GS1; (**c**) PVA/GS2; (**d**) PVA/GLY; (**e**) PVA/GS1/GLY; (**f**) PVA/GS2/GLY; and (**g**) PVA/GS1 ex situ.

Cross-sectional SEM images of unplasticized and glycerol plasticized PVA and PVA/GS nanocomposites prepared by in situ reduction of GO with L-AA, and by the ex situ process are shown in Figure 4. SEM micrographs of PVA (Figure 4a,d) have a clean and smooth surface. From Figure 4b–f, it can be observed that the images of PVA/GS, prepared by in situ reduction, with 1.0 and 2.0 wt% GS content exhibit a much rougher fractured surface with no aggregates of GS, and a wave-like morphology. The roughness of the fracture surface increases when plasticizer is present. This result indicates a uniform dispersion of GS in PVA matrix. The rougher fracture surface is attributed to the interfacial adhesion and compatibility between polymer matrix and graphene nanosheets. However, the micrograph of the nanocomposite obtained by the ex situ method (Figure 4g) shows a cleaner and smoother surface than its in situ obtained counterpart, indicative of poor dispersion of GS due to the lack of interfacial interaction and adhesion of GS to PVA.

**Figure 5:**
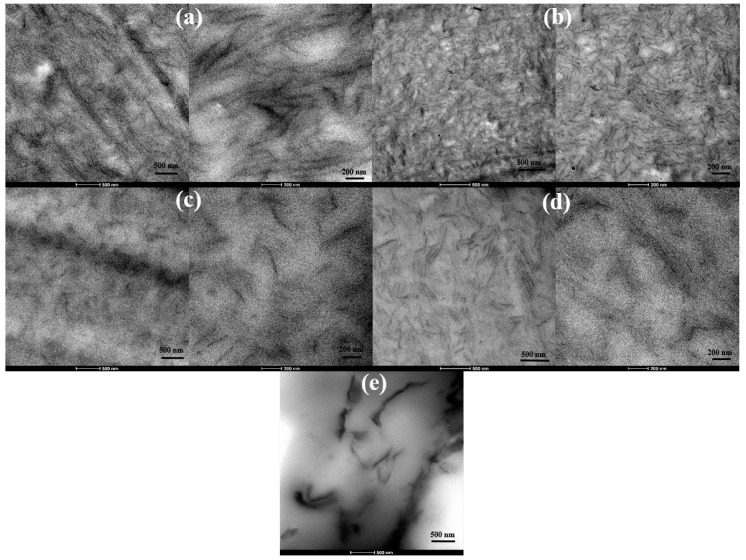
TEM images of: unplasticized (**a**) PVA/GS1, (**b**) PVA/GS2; plasticized (**c**) PVA/GS1, (**d**) PVA/GS2; and (**e**) PVA/GS1 ex situ, at different magnifications.

TEM images of unplasticized and glycerol plasticized PVA/GS nanocomposites with 1 and 2 wt% GS presented in Figure 5a–d evidence the good dispersion state of graphene sheets throughout PVA. Single dispersed sheets and aggregated nanosheets with thickness ~12 nm coexist. However, a better degree of dispersion is achieved in the plasticized nanocomposites. From TEM and SEM analysis, and XRD patterns it can be inferred an exfoliated morphology for the samples prepared by the in situ method. For the nanocomposite sample obtained by the ex situ method, a poor dispersion and exfoliation of graphene sheets is observed (Figure 5e).

#### 3.2.3. Thermal Properties

##### Differential Scanning Calorimetry (DSC)

**Figure 6:**
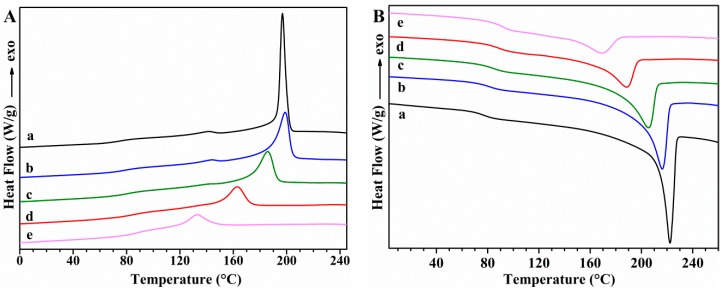
DSC curves: (**A**) first cooling; (**B**) second heating for unplasticized (a) PVA; (b) PVA/GS0.5; (c) PVA/GS1; and (d) PVA/GS1.5; (e) PVA/GS2.

**Figure 7:**
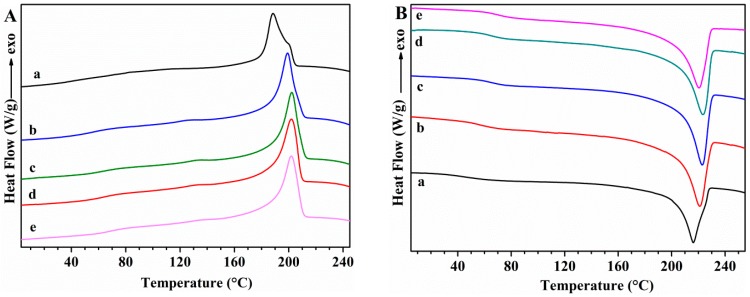
DSC curves: (**A**) first cooling; (**B**) second heating for (a) PVA/GLY; (b) PVA/GS0.5/GLY; (c) PVA/GS1/GLY; (d) PVA/GS1.5/GLY; and (e) PVA/GS2/GLY.

The thermal transitions of PVA samples and the effect of GS incorporation on these transitions were studied using DSC. First cooling and second heating scans for unplasticized PVA and PVA/GS nanocomposites are shown in Figure 6, while Figure 7 shows those for glycerol plasticized PVA and PVA/GS nanocomposites. The DSC results for these samples are summarized in Table 1.

The DSC cooling thermograms of neat unplasticized and plasticized PVA (Figure 6(Aa) and Figure 7(Aa)) show an exotherm at 197.7 °C and 188.7 °C, respectively, due to the crystallization. The presence of plasticizer causes the lowering of the crystallization temperature (*T*_c_) since it forms hydrogen bonds with PVA and acts as a diluent for the PVA chains [64]. The *T*_c_ is almost unaffected by the incorporation of 0.5 wt% of GS to unplasticized PVA and a further increase of GS content leads to lower *T*_c_ values, indicating that crystallization is retarded. *T*_c_ value of plasticized PVA increases by adding 0.5 wt% of GS, is almost unaffected by the presence of 1 wt% of GS, whilst increases with a further increase in GS loading. At a loading of 0.5 wt% GS the nanofiller may act as nucleating agent which results in a higher *T*_c_ value, while the lower *T*_c_ values attained at GS loadings higher than 0.5 wt% are attributed to the hydrogen bonding between PVA and GS. The *T*_c_ value of the blend of PVA with 1 wt% of GS prepared by the ex situ method is almost unaffected as compared with that of neat PVA, and 14 °C higher than the value of the sample prepared by in situ process.

In the second heating (Figure 6B), unplasticized PVA has an endothermic peak at 221.7 °C that corresponds to the melt of crystalline phase of PVA with a heat of fusion of 76.16 J g^−1^, whereas when PVA is plasticized with glycerol (Figure 7Ba) the endotherm broadens and shifts toward lower temperature, at 216 °C, and the heat of fusion (49.78 J g^−1^) significantly diminishes. This variation in *T*_m_ and enthalpy of fusion values is attributed to the hydrogen-bonding interaction between the glycerol and PVA, the inter- and intra-hydrogen bonds in PVA chains are weakened and the molecular motions are eased [64]. After incorporation of different amounts of GS, in both unplasticized and glycerol plasticized PVA, important changes are observed in the *T*_m_ values as compared with neat PVA (Table 1). *T*_m_ decreases gradually as the GS content in unplasticized PVA increases, and the same trend is observed in the case of the enthalpy of fusion. As for plasticized PVA *T*_m_ value is almost unaffected by the incorporation of 0.5 wt% of GS, whereas the enthalpy of fusion increases. GS contents higher than 0.5 wt% cause a lowering in the *T*_m_ and Δ*H*_m_ values. This behavior is attributed to the interaction between GS and PVA. These results are consistent with those found in previous studies on PVA/reduced GO nanocomposites obtained by reducing GO by hydrazine in the presence of PVA, ref. [40] although in our study the changes in *T*_c_ and *T*_m_ are significantly higher.

The percentage crystallinity (% *X*_c_) of PVA was determined using the following equation:

(4) $$X_{C}=\left[\frac{\Delta H_{m}}{\Delta H_{m}^{0}\times \left(1-\frac{\%{\mathrm{wt}}_{filler}}{100}\right)}\right]\times 100$$

where Δ*H*_m_ is the heat of fusion of the PVA and PVA/GS nanocomposites and $\Delta H_{m}^{0}$ is the heat of fusion of the 100% crystalline PVA (141.932 J g^−1^) [65], and wt% filler is the total weight percentage of GS. The crystallinity decreases after plasticization of PVA with 20 wt% glycerol, from 53.7 to 35.1%. The incorporation of GS also causes a remarkable decrease in the degree of crystallinity of unplasticized PVA, from ~54% for PVA to 15% for the sample containing 2 wt% GS, whereas in the case of plasticized PVA the incorporation of 0.5 wt% of GS leads to a slight increase in crystallinity and then a higher amount of GS causes a decreasement. This reduction of the crystallinity of PVA in the nanocomposites, that can be explained by the formation of a constrained polymer region [62], indicates some interaction between the polymer chains and the filler. The crystallinity changes induced by the incorporation of GS are in good agreement with the XRD results and previous studies on PVA/graphene nanocomposites synthesized by reducing GO with hydrazine in the presence of the polymer matrix [40].

The glass transition temperature (*T*_g_) of neat PVA decreases upon addition of glycerol from 76.1 °C to 42.5 °C, indicating and enhancement of chain segment mobility. Glycerol destroys the inter- and intra-molecular hydrogen bonds in PVA chains, facilitating the molecular motions of polymer chains. When GS is incorporated both in unplasticized and in glycerol plasticized PVA, *T*_g_ increases gradually as the nanofiller content increases, as a result of the hydrogen bonding interactions between GS and PVA [66]. The interfacial interactions between GS and PVA make the polymer matrix more rigid. Similar results have been reported by other authors in their studies on PVA/graphene nanocomposites prepared by in situ reduction of GO [35,40]. Plasticized PVA/GS nanocomposites show a larger *T*_g_ increase than their unplasticized counterparts, indicating that there are more interactions in the presence of plasticizer.

*T*_m_, Δ*H*_m_, degree of crystallinity and *T*_g_ were not affected upon the incorporation of previously prepared GS to PVA (Table 1), indicating that there are not enough interactions between graphene and polymer chains to change the thermal behavior of PVA if the film is prepared by the ex situ method.

##### Thermogravimetric Analysis (TGA)

**Figure 8:**
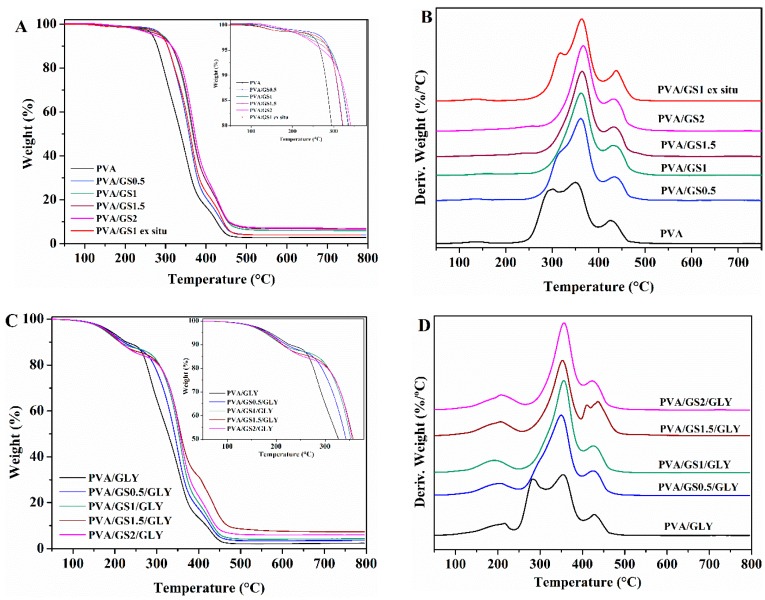
TG and DTG curves: (**A**,**B**) unplasticized, and (**C**,**D**) glycerol plasticized PVA and PVA/GS nanocomposites in N_2_.

**Figure 9:**
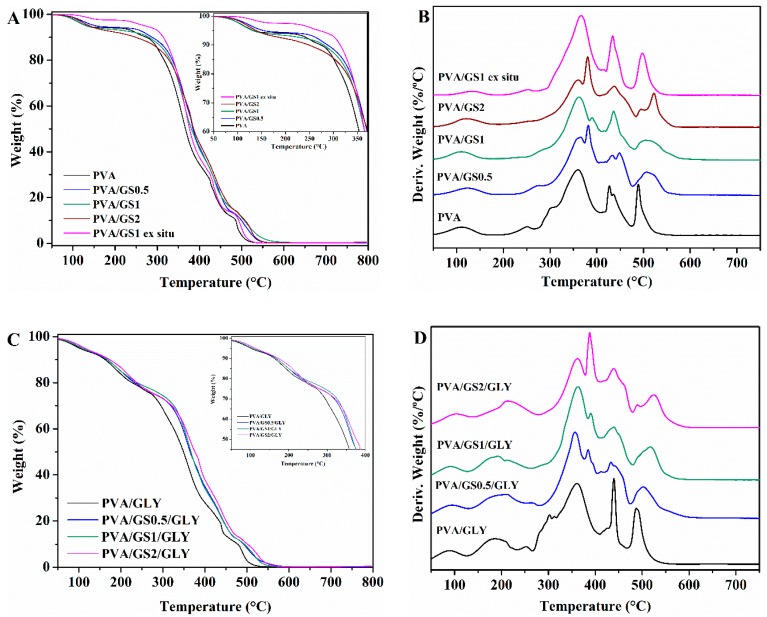
TG and DTG curves: (**A**,**B**) unplasticized, and (**C**,**D**) glycerol plasticized PVA and PVA/GS nanocomposites in air.

The effect of GS and glycerol on the thermal and thermoxidative stability of PVA films was evaluated by TGA. Thermogravimetric and differential thermogravimetric curves for PVA, glycerol-plasticized PVA, and their GS nanocomposites are shown in Figure 8 and Figure 9, and the characteristics of thermal degradation *T*_5%_ and *T*_50%_ (temperatures corresponding to 5% and 50% weight loss) and the fraction of solid residue at 800 °C summarized in Table 2. The TG and DTG curves of PVA in nitrogen atmosphere show that it degrades in two steps in the temperature range of 200 to 500 °C. The main degradation stage occurs between 200 °C and 400 °C with a weight loss of 81%. The residue remaining at 800 °C is about 3%. In the first stage side groups (OH) are eliminated from PVA and chain scission reactions take place, while in the second one the breakdown of the polymer backbone occurs [67,68].

The presence of GS has effect on the degradation of PVA, there is a significant change in the shape of the DTG curve of PVA/GS composites as compared with that of neat PVA (Figure 8B). the first stage of decomposition of PVA exhibits two peaks on DTG curve (Figure 8B), whereas, after incorporation of GS the first peak disappears, and the intensity of the second peak increases, that is, the two peaks merge together (at GS contents higher than 0.5 wt%) or closely to one (at 0.5 wt% GS). The dehydration reaction is slow down in the presence of GS. The *T*_5%_ and *T*_50%_ increases with the addition of GS, those values for the nanocomposites with 0.5 wt% and 1 wt% GS increases by 20 and 30 °C, respectively, when compared with neat PVA. The nanocomposite containing 1 wt% GS exhibits the highest thermal stability. The PVA/GS nanocomposites leave higher char residue at 800 °C as compared with neat PVA. In the case of the nanocomposite PVA/GS1 prepared by the ex situ method, the *T*_5%_ and *T*_50%_ values are higher than those of neat PVA but slightly lower than the values for the in situ prepared sample. In the DTG curve of the blend prepared by the ex situ method, unlike what had happened with the blend prepared by in situ method, the first peak due to the side groups elimination does not disappear, although its intensity is reduced.

The TGA and DTG profiles of PVA and plasticized PVA are similar, except that a new step appears in the presence of glycerol, between 100 °C and 235 °C, which corresponds to the evaporation of the plasticizer. The thermal stability of plasticized PVA is slightly lower than neat PVA. The *T*_5%_ and *T*_50%_ increases with the addition of GS to plasticized PVA, the *T*_5%_ value of the nanocomposite with 2 wt% GS increases by 45 °C when compared with neat PVA. The higher thermal stability of graphene filled PVA can be attributed to the high thermal stability of GS, to the mass transport barrier effect of uniformly dispersed graphene sheets to volatile degradation products [69], and also to char formation. The diffusion of volatile gas evolved during the thermal decomposition is hindered, the oxygen diffusion into the polymer matrix is prevented. The presence of GS in the PVA matrix inhibits the side groups elimination due to the absorption of free-radicals generated during polymer decomposition by the carbon surface [70], to the interaction of oxygen functionalities of GS with –OH groups of PVA and to the tortuous path formed by the GS homogeneously distributed in the PVA matrix, that prevents the gas scape. In the case of the sample prepared by the ex situ method the distribution is non uniform and the interfacial interactions are weaker.

The decomposition process in air atmosphere (Figure 9) differs from that under inert environment. The thermogram of PVA reveals, in addition to the loss of physically adsorbed water around 100 °C, another four steps. The first stage with maximum rate at 300 °C assigned to the partial dehydration of polymer chains followed by the polyene formation, and the second one with maximum rate at 360 °C attributed to the polyene decomposition to form macroradicals [71]. The third stage with maximum rate at 427 °C is the result of intramolecular cyclization and condensation of polyconjugated aromatic structures formed from the decomposition of polyene macroradicals, and the last step with maximum rate at 489 °C is due to the thermo-oxidation of carbonized residue [71].

As in the case of inert atmosphere, the plasticized PVA exhibits another decomposition step between 150 °C and 250 °C due to the glycerol evaporation. The *T*_5%_ and *T*_50%_ of both unplasticized and glycerol plasticized PVA/GS nanocomposites are higher than neat PVA. Therefore, GS increases the thermo-oxidative stability of PVA. The enhancement of thermal stability can be attributed to the physical protective barrier of GS in the PVA matrix, retarding the escape of volatile degradation products. Besides blocking the dehydration of polymer, GS retards the thermo-oxidation of carbonized residue (Figure 9B–D) which can be attributed to the barrier effect of GS that makes it more difficult for oxygen to reach the polymer.

#### 3.2.4. Mechanical Properties

**Figure 10:**
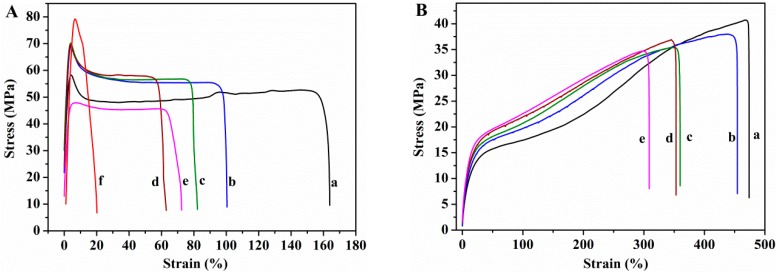
Stress–strain curves of (**A**) unplasticized and (**B**) glycerol plasticized: (a) PVA; (b) PVA/GS0.5; (c) PVA/GS1; (d) PVA/GS1.5; (e) PVA/GS2; and (f) PVA/GS1 ex situ.

**Figure 11:**
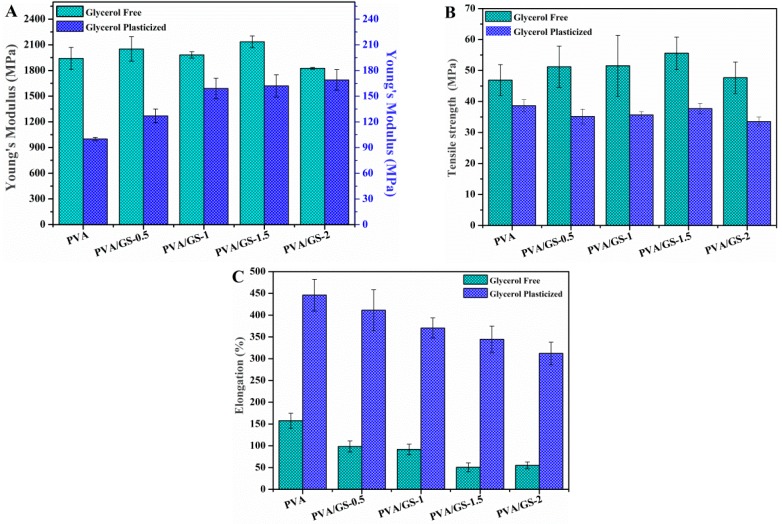
Mechanical properties of PVA/GS nanocomposite films: (**A**) Young’s modulus; (**B**) tensile strength at break; and (**C**) elongation at break with various amounts of GS.

Tensile tests were used to determine the mechanical properties. The Young’s modulus, tensile strength at break, and the elongation at break measured from the stress–strain curves (Figure 10) are shown in Figure 11.

Glycerol plays an important role in the mechanical properties of PVA films. The Young’s modulus and tensile strength of the PVA film decrease (95% and 18%, respectively) upon incorporation of glycerol, whereas the elongation increases enormously, 183%, indicating that PVA film becomes softer and more ductile. Glycerol destroys the inter and intra-molecular hydrogen bonds in PVA chains, facilitating the molecular motions of polymer chains, making PVA films more flexible. Changes in the degree of crystallinity due to the presence of plasticizer must also be taken into account. The decrease in the Young’s modulus and tensile strength can be ascribed to the lower degree of crystallinity of plasticized PVA with respect to the unplasticized polymer. The presence of GS has no effect on the elastic modulus of unplasticized PVA, the changes in the Young’s modulus found for the unplasticized nanocomposite films lie within the experimental error. However, in the case of plasticized nanocomposites the modulus increases as GS content increases, the maximum increasement attained is 70% at 2 wt% GS loading. The reinforcing action of GS is stronger in the presence of glycerol due to the better interfacial contact of the filler with PVA matrix, which is in good agreement with SEM and TEM results.

The tensile strength of the unplasticized nanocomposite films increases slightly up to 1.5 wt% GS content, from 47 to 56 MPa. In the case of the plasticized films, the tensile strength remains almost constant after incorporation of GS. The elongation at break value of both unplasticized and plasticized films decreases as the GS content increases, being the reduction higher in the case of the unplasticized nanocomposites, 65% for the unplasticized film containing 2 wt% GS and 30% for the glycerol plasticized counterpart, indicating that the graphene increases the brittleness of the films. The degree of crystallinity is an important parameter for semi-crystalline polymers that has effect on their mechanical properties. PVA crystallinity decreases as GS content increases, however, the reduction in the degree of crystallinity has not drastically affected the mechanical behavior of PVA/GS nanocomposites, which can be attributed to the interfacial interaction between PVA and graphene. The enhancement of mechanical properties in nanocomposite films of PVA/GS prepared by in situ reduction of GO has been reported in the literature. Zhao et al. [36], Yang et al. [38], and Bao et al. [41] reported increase in the Young’s modulus and the tensile strength and decrease in the elongation at break of up to 90%.

The value of the modulus of elasticity of the nanocomposite containing 1 wt% GS prepared by the ex situ method is similar to that of PVA. The tensile strength value (67.7 ± 7.2 MPa), implies an increase of 44% with respect to the value for neat PVA, whereas the elongation at break decreases drastically from 158 to 12%, implying a decrease of 92% with respect to the value for neat PVA. In the case of this film, it must be taken into account that the degree of crystallinity remains unchanged as compared with neat PVA. The lower elongation at break of the PVA/graphene nanocomposites compared to neat PVA indicates that the incorporation of GS into PVA increases the brittleness of the composite, especially in the case of the unplasticized composite prepared by the ex situ method. This can be due to the poorer dispersion of GS in PVA matrix as compared with the nanocomposite film prepared by in situ process.

#### 3.2.5. Water Absorption and Water Vapor Permeability

**Figure 12:**
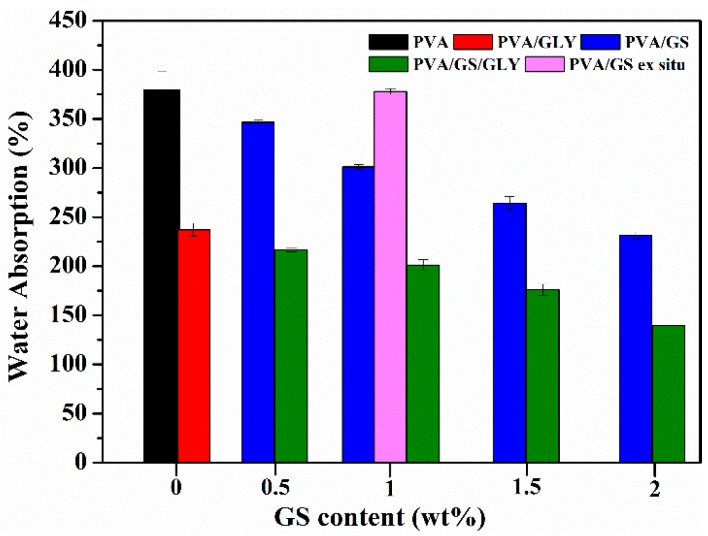
Water absorption for unplasticized and glycerol plasticized PVA and PVA/GS nanocomposites.

In Figure 12 the total water absorbed by the unplasticized and glycerol plasticized PVA and PVA/GS nanocomposite films is plotted against GS content. Neat unplasticized PVA film exhibits the highest water absorption value, whilst in the presence of glycerol a significantly reduction in the water uptake (~42%) is observed. This reduction can be attributed to the hydrogen bonding between glycerol and PVA, the polar groups of PVA form hydrogen bonds with hydroxyl groups of plasticizer and they are unable to fix water molecules, and therefore the insertion of water molecules is hindered. The incorporation of GS into unplasticized and glycerol plasticized PVA by in situ method leads to a reduction in the amount of water uptake. The water absorption of PVA/GS nanocomposites decreases by 8.8%, 20.7%, 30.4%, and 39.4% after incorporation of 0.5 wt%, 1 wt%, 1.5 wt%, and 2 wt% of GS, respectively, when compared with neat PVA. Similarly, the incorporation of those amounts of GS to glycerol plasticized PVA decreases the water absorption by 8.6%, 15.1%, 25.7%, and 41.1%, respectively. The water absorption value of the unplasticized nanocomposite containing 1 wt% GS prepared by the ex situ method is similar to that of neat PVA film. The lower water absorption of nanocomposite films as compared with neat PVA can also be explained by the formation of the constrained polymer region [62]. The reduced mobility of the polymer chains in this region as a result of the interfacial adhesion between GS and PVA inhibits the insertion of water molecules. The lower water uptake of the film prepared by the in situ method as compared with the ex situ counterpart should result from the stronger interfacial interaction between GS and PVA when it is incorporated by the in situ process.

**Figure 13:**
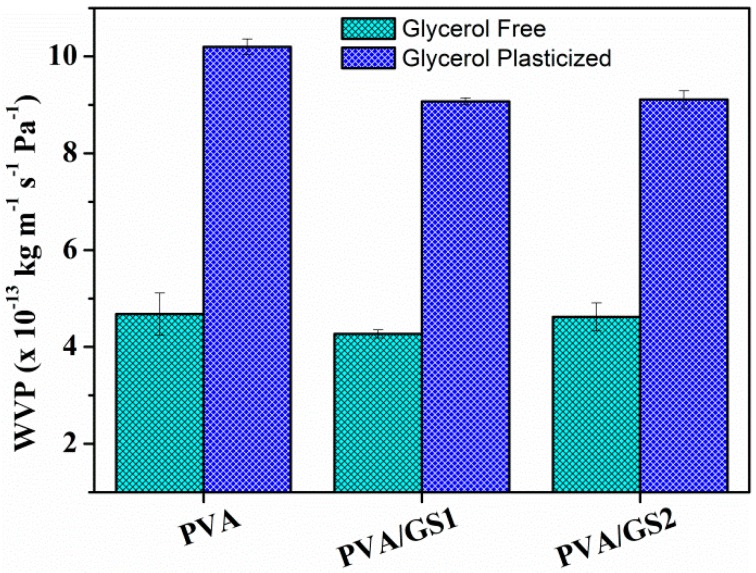
Water vapor permeability (WVP) for unplasticized and glycerol plasticized PVA and PVA/GS nanocomposites.

Water vapor permeability (WVP) of unplasticized and plasticized PVA/GS nanocomposites with different contents of GS was measured at a vapor pressure difference of 100/58% RH (i.e., at a RH gradient of 100/58) across the film. WVP data are shown in Figure 13.

The WVP of PVA increases, about 120%, with the addition of glycerol. This can be ascribed to the hydrophilic nature of glycerol, its hydroxyl functions interact with the hydroxyl groups of PVA, decreasing the intermolecular attractions along the PVA chains and, consequently, the chain mobility increases thus facilitating the water vapor diffusivity and through the PVA film and accelerating the water vapor transmission [72]. The incorporation of GS (1 wt%) to both plasticized and unplasticized PVA leads to about 10% reduction in WVP values with respect to neat polymer. No important changes are observed as a function of GS content. The reduction in PVA film permeability can be due to the more tortuous path that water molecules require to permeate through the polymeric matrix because of the presence of graphene sheets distributed in PVA, leading to slower diffusion process and to lower WVP. The small reduction of WVP upon incorporation of GS can be attributed to the effect of crystallinity on the solubility of water vapor in polymers. PVA/GS nanocomposites exhibit lower crystallinity (Figure 3) than neat PVA, and free amorphous regions exhibit lower resistance to water vapor permeation than crystalline regions [73].

PVA has been widely researched as biomaterial for a variety of biomedical and pharmaceutical applications [5], including in transdermal drug delivery [74,75,76]. The mechanical properties of the materials are important for the potential application in this field. A suitable transdermal film should have a relatively high tensile strength, since it has to withstand the rupture, and high elongation at break, that is, the film must be flexible since it must not be torn by external forces, such as folding, stretching, and peeling off the surface. Permeability to water vapor of the film is another important characteristic as it has influence on skin properties like hydration. Good water vapor permeation is necessary to avoid skin maceration, especially when the patch remains in the same position for prolonged period of time [77]. On the other hand, owing to the unique properties of graphene it has a great potential in biomedical applications, including carriers for drug delivery [78,79,80,81,82]. Biocompatibility and cytotoxicity are critical aspects for the successful application of any material in biomedical and pharmaceutical fields. Results based on in vitro and in vivo evaluation of the cytotoxicity and biocompatibility of graphene-based materials revealed that physiochemical properties such shape, size and distribution, surface charge, surface area, layer number, lateral dimensions, surface chemistry, purity, particulate state, surface functional groups, synthesis methods, route, and dose of administration, and exposure times have effect on their toxicity [83,84]. Li et al. [85] evaluated the cytotoxicity of PVA/GO films directly reduced with hydroiodic acid and found that the films were non-cytotoxic. Glycerol is nontoxic plasticizer for food and biomedical applications, biodegradable, biocompatible, environmental friendly, and widely used in pharmaceutical applications [86,87]. Glycerol is harmless to the skin. On the basis of the above analysis, and taking into account the mechanical properties, the water absorption, and the water vapor permeability of the nanocomposite films prepared in our study, PVA/GS/glycerol nanocomposites could be considered the most suitable materials for the above-mentioned application.

## 4. Conclusions

The in situ reduction of GO in the presence of PVA by L-AA strategy adopted in this work to obtain PVA/GS nanocomposites resulted in a good dispersion state of GS in the polymer matrix due to interactions through hydrogen bonding. The GS content and the presence of glycerol as a plasticizer affected the thermal transitions (*T*_g_, *T*_m_ and *T*_c_) and the degree of crystallinity. On the contrary, no change was observed when the nanocomposite was obtained by ex situ reduction of GO before the composite preparation as a result of a poor dispersion and weak bonding between GS and PVA. GS exerted a blocking effect on the elimination of hydroxyl groups of PVA chains during thermal decomposition, delaying its thermal and thermo-oxidative degradation. The mechanical properties of the PVA were differently affected by the presence of GS depending on whether plasticizer was or not present. The mechanical properties of nanocomposites were not drastically affected by the reduction in PVA crystallinity. No variation on Young’s modulus was observed in the unplasticized nanocomposites, whereas it increased (70% at 2 wt% GS) in the plasticized films, the reverse trend was found in the tensile strength. The nanocomposites were more brittle than neat PVA, the most remarkable reduction in elongation at break was observed in the ex situ-prepared nanocomposite film. A significant reduction of water absorption by the PVA occurred in the presence of GS as a result of the interfacial interactions, whereas no change was observed for the ex situ prepared PVA/GS nanocomposite. A slight reduction in WVP of PVA/GS nanocomposites was noticed due to the lower crystallinity of the nanocomposite films as compared with neat PVA. Owing to the improvements on properties of PVA after incorporation of GS as a result of the interfacial interactions between the residual oxygenated groups on GS and hydroxyl groups of the polymer chains, these nanocomposites, especially the plasticized films could be candidate for biomedical applications as transdermal drug delivery.

## Figures and Tables

**Table 1:** DSC data for unplasticized and plasticized PVA and PVA/GS nanocomposites.

Sample	*T*_g_ (°C)	*T*_m_ (°C)	Δ*H*_m_ (J/g)	*T*_c_ (°C)	Δ*H*_c_(J/g)	*X*_c_ (%)
PVA	76.1	221.7	76.2	197.7	66.5	53.7
PVA/GS-0.5	80.3	215.8	67.9	199.2	51.6	48.1
PVA/GS-1	84.4	205.2	53.8	185.9	39.8	38.3
PVA/GS-1.5	88.0	188.2	34.1	163.0	28.3	24.4
PVA/GS-2	91.9	169.2	21.0	133.0	21.6	15.1
PVA/GS-1 ex situ	75.0	221.9	75.7	200.1	60.1	53.9
PVA/GLY	42.5	216.0	49.8	188.7	47.7	35.1
PVA/GS-0.5/GLY	72.0	218.3	58.8	199.6	50.1	41.6
PVA/GS-1/GLY	77.0	205.5	48.8	189.5	39.2	34.7
PVA/GS-1.5/GLY	73.6	196.6	33.8	176.8	28.2	24.2
PVA/GS-2/GLY	83.6	187.9	31.7	167.6	21.1	22.8

**Table 2:** TGA data for unplasticized and plasticized PVA and PVA/GS nanocomposites.

Sample	*T*_5%_ (°C)		*T*_50%_ (°C)		Residue (%)
	N_2_	O_2_	N_2_	O_2_	N_2_
PVA	271	281	338	367	2.9
PVA/GS-0.5	293	296	358	384	4.1
PVA/GS-1	300	291	367	381	6.0
PVA/GS-1.5	295		368		6.6
PVA/GS-2	287	284	372	386	7.1
PVA/GS-1 ex situ	295	303	361	376	4.1
PVA/GLY	266	272	327	349	2.5
PVA/GS-0.5/GLY	286	317	342	365	3.7
PVA/GS-1/GLY	298	291	352	367	4.3
PVA/GS-1.5/GLY	302		358		7.3
PVA/GS-2/GLY	310	311	355	377	6.0

## Supplementary Materials

The following are available online at http://www.mdpi.com/2079-4991/8/12/1013/s1, Figure S1: Infrared spectra of natural graphite, GO, and GS, Figure S2: XRD patterns of natural graphite, GO, and GS, Figure S3: Microscopy images of GS: (a) SEM image, (b) TEM image, Figure S4: TGA and DTG curves of natural graphite, GO, and GS, Figure S5: FTIR spectra of (a) PVA, (b) PVA/GS1, (c) PVA/GS2, (d) PVA/GLY, (e) PVA/GS1/GLY, (f) PVA/GS2/GLY.
