# Supplementary material for: Graphene Based Poly(Vinyl Alcohol) Nanocomposites Prepared by In Situ Green Reduction of Graphene Oxide by Ascorbic Acid: Influence of Graphene Content and Glycerol Plasticizer on Properties
Source: Nanomaterials (Basel). 2018 Dec 6;8(12):1013. doi: 10.3390/nano8121013 (PMC6316035; doi:10.3390/nano8121013)
Supplement: Supplementary file 1 [file nanomaterials-08-01013-s001.pdf]

Supplementary Materials

Graphene based Poly(Vinyl Alcohol) Nanocomposites Prepared by In Situ Green Reduction of Graphene Oxide by Ascorbic Acid: Influence of Graphene Content and Glycerol Plasticizer on Properties

Mónica Cobos, M. Jesús Fernández * and M. Dolores Fernández

Department of Polymer Science and Technology. Faculty of Chemistry, University of the Basque Country UPV/EHU, Paseo Manuel Lardizábal 3, 20018 San Sebastián, Spain; monica.cobos@ehu.es (M.C.); mariadolores.fernandez@ehu.es (M.D.F.)

* Correspondence: mjesus.fernandez@ehu.es; Tel.: +34-943-015353

1. Experimental

1.1. Methods for the characterization of GO, GS and PVA/GS nanocomposites

FTIR spectroscopy was employed to examine the chemical structure of GO, GS and PVA/GS nanocomposites. The spectra were acquired using Nicolet iS10 spectrometer from pressed KBr pellets loaded with the graphenic materials, while the spectra of PVA and PVA/GS films were recorded with attenuated total reflectance (ATR) accessory.

SEM images of GS were obtained using a Hitachi S-4800 scanning electron microscope at an accelerating voltage of 15 kV. TEM micrographs of the GS were obtained with a Philips Tecnai G2 20 TWIN TEM at 200 kV accelerated voltage. TGA measurement of graphenic materials was performed under nitrogen atmosphere on a TA instruments TG-Q-500 at a heating rate of 10 °C/min from 40 °C to 800 °C.

2. Results and Discussion

2.1. Characterization of GO and GS

The formation of GO was proved by FTIR analysis, and its chemical composition was measured by XPS. FTIR spectrum of GO exhibits new peaks compared to that of natural graphite (Figure S1), the bands in the 3800–3000 cm^{-1} range, attributed to the stretching vibrations of structural OH groups and physisorbed water molecules; the peaks at 1734 cm^{-1} and 1362 cm^{-1}, assigned to the C=O carbonyl stretching of COOH groups situated at the edges of the oxidized graphenes and to the bending of tertiary C–OH groups, respectively; and the peaks at 1052 cm^{-1} and 980 cm^{-1} attributed to the vibration of C–O of epoxide groups (C–O–C) and to epoxide or peroxide groups, respectively. Thus, the presence of epoxy, –C=O and –OH groups in GO is confirmed by FTIR data. In the spectrum of GS the band due to the intercalated water at 3417 cm^{-1} is narrower than in the spectrum of GO, since in the last case two peaks contribute to the signal, the peaks associated to hydroxyl and –COOH groups at 3584 cm^{-1} and 3194 cm^{-1}. In addition, the weakening of the bands ascribed to carbonyl stretching, epoxides and hydroxyls, and the disappearance of the band at 1621 cm^{-1} can be observed in the spectrum of GS. After reduction, two new bands at 1558 cm^{-1} (aromatic C=C stretching vibration) and 1117 cm^{-1} (aromatic C–H in-plane bending vibration) evidence the restoration of the sp^2 structure.

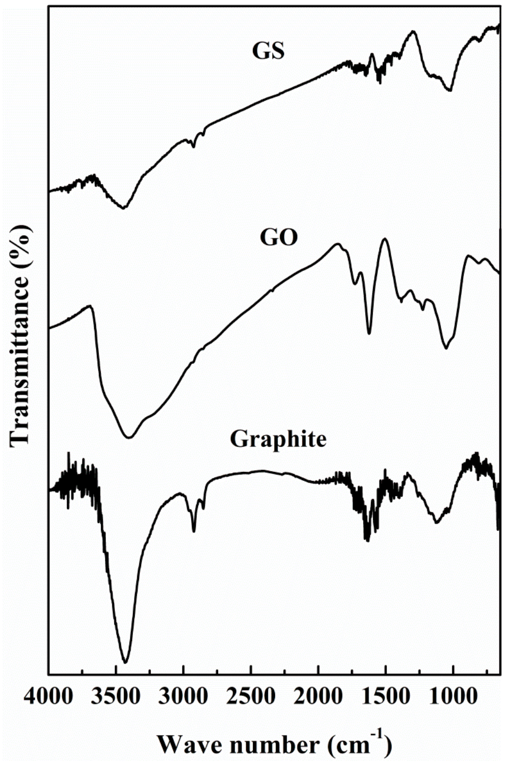

Figure S1. Infrared spectra of natural graphite, GO and GS.

The morphological characterization of graphenic materials (graphite, GO and GS) was investigated by XRD technique. In the XRD pattern of graphite (Figure S2) a sharp strong peak appears at 2θ = 26.7°, corresponding to the (002) diffraction line with the interlayer spacing along the c-axis of 0.33 nm [1,2]. After oxidation, a broad peak at 2θ= 11.4° appears, corresponding to *d* spacing of 0.78 nm, indicating that the crystal structure of natural graphite has been damaged during oxidatiion. This increasement in the interlayer spacing (*d*) is due to the introduction of epoxide and hydroxyl groups and the changes in the hybridization state of carbon atoms from sp^2 to sp^3 [3]. After chemical reduction the XRD pattern exhibits a broad peak (2θ= 24.3°; *d* spacing ~ 0.37 nm), indicating the exfoliation of chemically reduced graphene oxide into a few layers.

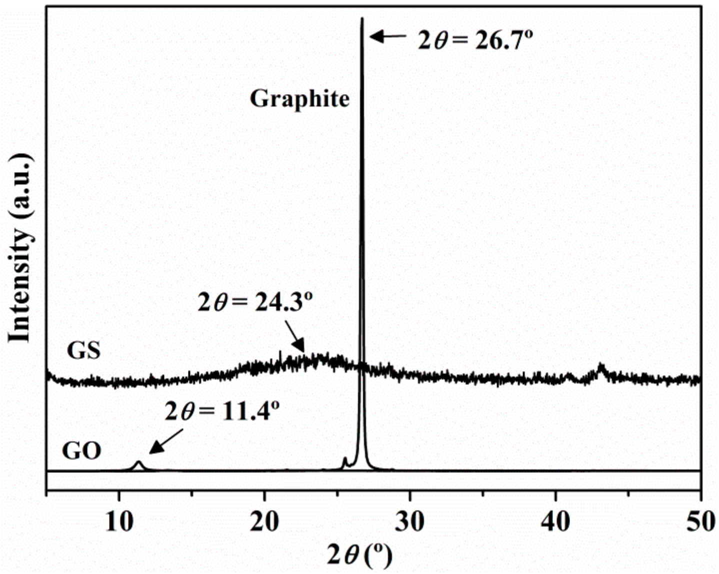

Figure S2. XRD patterns of natural graphite, GO and GS.

SEM and TEM was used to characterize the morphology and microstructure of GS. GS shows a layer-like structure and a crumpled morphology of randomly aggregated sheets (Figure S3). TEM image shows large graphene transparent curtain like sheets with black regions as a result of corrugation and scrolling of graphene sheets.

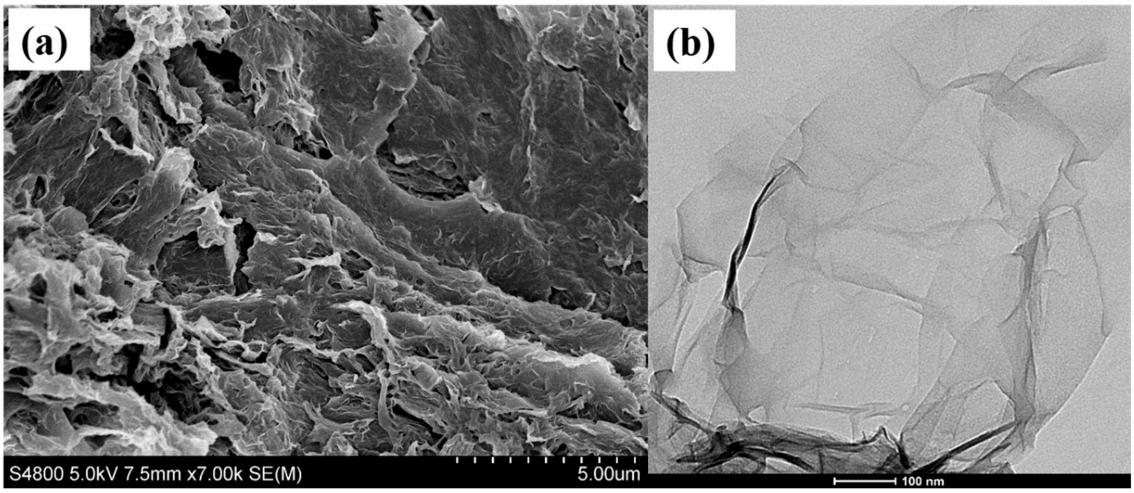

Figure S3. Microscopy images of GS: (**a**) SEM image, (**b**) TEM image.

TGA was used to determine the thermal stability of natural graphite, GO and GS, the reductive ability of L-AA, as well as to verify the accuracy of the C/O ratio data of GO and GS obtained by XPX spectroscopy (Figure S4).

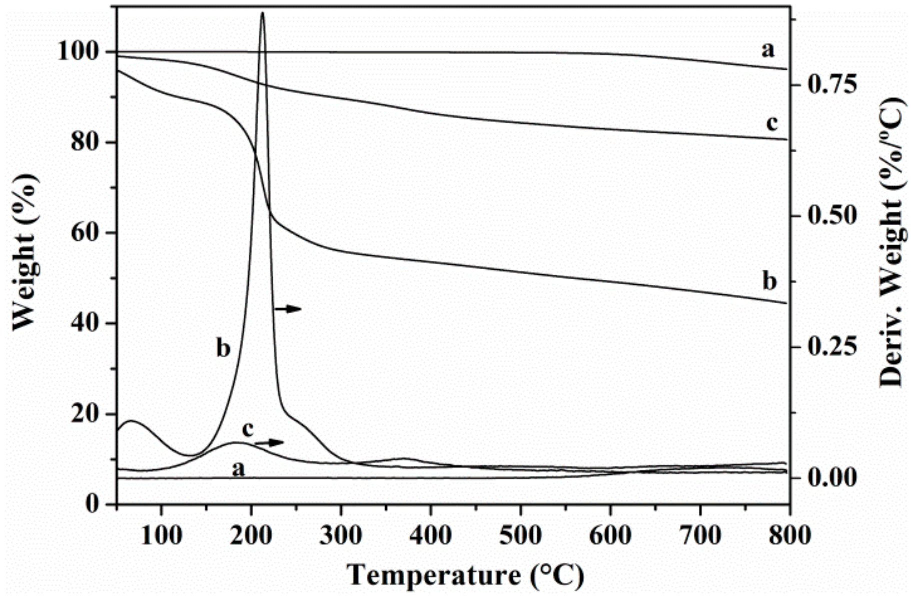

Figure S4. TGA and DTG curves of natural graphite, GO and GS.

Graphite decomposes in one step and shows high thermal stability up to 600 °C (Figure S4a). The thermal decomposition of GO takes place in two steps (Figure S4b), in the first one (50–120 °C) loosely bound or adsorbed water and gas molecules are eliminated. In the second one (125–300 °C) the decomposition of the most labile oxygen functionalities present in the material takes place, and its mass loss (32%) matches well with the value obtained by XPS (31.2 % of O). The removal of more stable oxygen functionalities and the decomposition of the carbon structure occurs in the temperature range above 300 °C [4,5]. Although the thermogram of GS (Figure S4c) is similar to that of GO, the second step shows lower weight loss (15 %), which can be explained by the removal of the oxygen-

containing groups from the GO surface during the chemical reduction. This result indicates that the oxygen functionalities are not totally absent in GS.

2.2. Characterization of PVA/GS nanocomposites

2.2.1. FTIR characterization

The FTIR spectrum of PVA (Figure S5a) exhibits the broad O–H stretch band, in the region 3650–3000 cm^{-1}, due to the intermolecular and intramolecular hydrogen bonds, the band between 2800 and 3000 cm^{-1} assigned to stretching of C–H from alkyl groups, the peaks from 1780–1600 cm^{-1} due to stretching of C═O and C–O from the residual acetate groups in PVA, and peaks attributed to the vibration of CH_2 (1418, 1326 cm^{-1}), CH (1237 cm^{-1}), and C–O (1087 cm^{-1}).

The FTIR spectrum of glycerol plasticized PVA shows an additional band at 1042 cm^{-1} characteristic of C–O stretching (Figure S5d), and the –OH stretching peak shifts to a higher wavenumber due to the hydrogen bonding between the hydroxyl groups of PVA and glycerol. In the FTIR spectra of unplasticized and glycerol plasticized PVA/GS nanocomposites (Figure 1b,c,e,f) it can be observed that the peak associated with OH stretching shifts to a higher wave number due to the hydrogen bonding with oxygenated groups of GS [6].

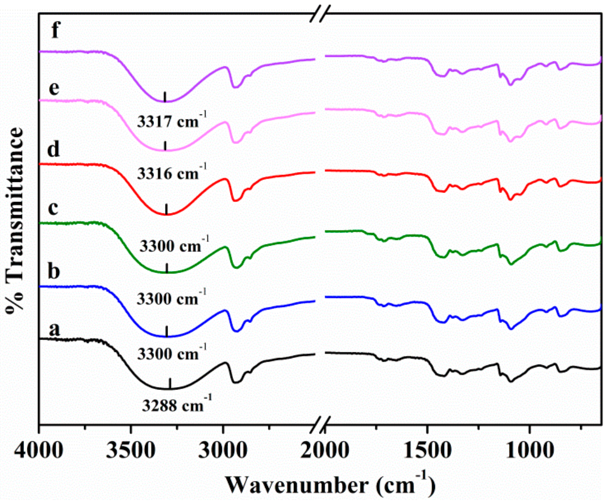

Figure S5. FTIR spectra of (a) PVA, (b) PVA/GS1, (c) PVA/GS2, (d) PVA/GLY, (e) PVA/GS1/GLY, (f) PVA/GS2/GLY.

References

1. Wang, G.; Yang, J.; Park, J.; Gou, X.; Wang, B.; Liu, H.; Yao, J. Facile synthesis and characterization of graphene nanosheets. *J. Phys. Chem. C.* **2008**, *112*, 8192–8195.
2. Park, S.; An, J.; Potts, J.R.; Velamakanni, A.; Murali, S.; Ruoff, R.S. Hydrazine-reduction of graphite and graphene oxide. *Carbon* **2011**, *49*, 3019–3023.
3. McAllister, M.J.; LiO, J.L.; Adamson, D.H.; Schniepp, H.C.; Abdala, A.A.; Liu, J.; Herrera-Alonso, M.; Milius, D.L.; Caro, R.; Prud'homme, R.K. Single sheet functionalized graphene by oxidation and thermal expansion of graphite. *Chem. Mater.* **2007**, *19*, 4396–4404.
4. Tang, X.Z.; Li, W.J.; Yu, Z.Z.; Rafiee, M.A.; Rafiee, J.; Yavari, F.; Koratkar, N. Enhanced thermal stability in graphene oxide covalently functionalized with 2-amino-4,6-didodecylamino-1,3,5-triazine. *Carbon* **2011**, *49*, 1258–1265.
5. Yang, H.F.; Li, F.H.; Shan, C.S.; Han, D.X.; Zhang, Q.X.; Niu, L.; Ivaska, A. Covalent functionalization of chemically converted graphene sheets via silane and its reinforcement. *J. Mater. Chem.* **2009**, *19*, 4632–4638.
6. Qi, X.; Yao, X.; Deng, S.; Zhou, T.; Fu. Q. Water-induced shape memory effect of graphene oxide reinforced polyvinyl alcohol nanocomposites. *J. Mater. Chem. A* **2014**, *2*, 2240–2249.
